# Laparoscopic lateral suspension for anterior and apical prolapse: a prospective cohort with standardized technique

**DOI:** 10.1007/s00192-021-04784-0

**Published:** 2021-04-09

**Authors:** Kyriaki Chatziioannidou, Nikolaus Veit-Rubin, Patrick Dällenbach

**Affiliations:** 1 Department of Pediatrics, Gynecology and Obstetrics, Division of Gynecology, Urogynecology Unit, Geneva University Hospitals, 30 boulevard de la Cluse, 1211, Genève 14, Switzerland; 2 Department of Obstetrics and Gynecology, Division of General Gynecology and Gynecologic Oncology, Urogynecology Unit, Medical University of Vienna, Vienna, Austria

**Keywords:** Pelvic organ prolapse surgery, Uterine prolapse, Apical prolapse, Sacrocolpopexy, Lateral suspension with mesh, Laparoscopy

## Abstract

**Introduction and hypothesis:**

Laparoscopic lateral suspension (LLS) for anterior and apical pelvic organ prolapse (POP) repair is a recent approach. Previous studies used various meshes or sutures. The purpose of this study was to evaluate outcomes of a standardized LLS technique.

**Methods:**

From January 2010 until December 2014, we performed POP repair by LLS with mesh on 88 women with anterior and apical POP ≥ stage 2. We used a polypropylene titanized mesh fixed to the vesico-vaginal fascia with absorbable sutures and treated posterior compartment defect by vaginal approach with native tissue repair if required. Between July 2013 and December 2018, all women were assessed by gynecological examination including the pelvic organ prolapse quantification (POP-Q) system. Subjective outcome was evaluated by the patient global impression of improvement (PGI-I) questionnaire.

**Results:**

Seventy-nine women (89.8%) were available for follow-up. The mean duration of follow-up was 3.4 years (SD 1.6). Mean age was 59.6 (SD 11.1) years and mean BMI 25.8 (SD 4.0) kg/m^2^. Ten patients (12.7%) had previous POP surgery. Fifty-two women (65.8%) required posterior colporraphy for associated posterior defect and 21 (26.6%) had associated urinary incontinence (UI) surgery. There were no perioperative complications. The objective cure rate (no prolapse beyond the hymen and no reoperation for POP recurrence) was 87.3%. The reoperation rate for recurrence was 5.1%. The subjective success rate (PGI ≤ 2) was 96.2%. There were no mesh exposures or extrusions.

**Conclusions:**

This standardized LLS is safe and effective with no mesh complications after 3-year follow-up.

## Introduction

Open abdominal and more recently laparoscopic sacrocolpopexy (LSCP) is considered the gold standard in the treatment of apical pelvic organ prolapse (POP) [1]. However, it is a difficult operation with potential complications. Promontory dissection may be challenging, especially in obese patients, with potential life-threatening vascular injuries and hypogastric nerve impairment [2]. In two large series, bladder and bowel functions were impaired after LSCP. De novo stress urinary incontinence (SUI) was described in 24.2%, respectively, in 46% of women, and constipation in 18.8% and 45% after LSCP [3, 4]. There was also 3% rectal injury in one of these series [3]. The rate of reoperation for mesh-related complications after LSCP was close to 3% in a recent large series [5]. In 1967, Kapandji described a technique of lateral suspension to the iliac bone during laparotomy [6]. In the 1990s, the technique was further adapted for laparoscopy. Lateral laparoscopic suspension (LLS) of apical POP with mesh makes it possible to avoid dissection of the promontory thus reducing the risks described before [7–10]. Previous LLS studies showed promising results but they were not standardized. Surgeons used various prosthetic materials, permanent sutures or glue to fix the mesh to the vesicovaginal fascia, with short-term follow-up. Vaginal mesh exposure or extrusion rate was close to 6% [8, 9]. Posterior compartment prolapse was treated with either a posterior mesh or standard colporraphy. A recent Cochrane review confirmed that the posterior compartment is best treated by the vaginal approach [11]. In a previous study we showed that using the appropriate mesh material and avoiding use of prosthetic material in the posterior compartment may reduce the risk of vaginal mesh exposure [12]. We hence decided to standardize the technique by always using the same polypropylene titanized mesh fixed to the vesico-vaginal fascia only with absorbable stitches and by treating posterior compartment defect by the vaginal approach with native tissue repair if required. Our hypothesis was that it is the inflammatory reaction mediated by the mesh which is responsible for the long term support and not the permanent sutures used by most authors during laparoscopic treatment of POP. Permanent sutures might conduct bacteria to the mesh and thus favor erosion. We believed this method would reduce the vaginal mesh exposure rate while maintaining the success of the technique.

The primary objective of this study was to analyze long-term objective and subjective outcomes of a standardized LLS with mesh in the treatment of POP. A secondary objective was to analyze long-term complications, in particular mesh-related complication rate.

## Materials and methods

We conducted a prospective observational clinical study. This study was approved by the Institutional Ethics committee of Geneva University Hospitals (protocol number 13-054). All patients gave their informed consent. From January 2010 until December 2014, we performed LLS POP repair with mesh in 88 consecutive women suffering from symptomatic POP ≥ stage 2 including apical and anterior vaginal wall defect. All patients had a standardized preoperative POP assessment using the POP-Q system [13] and preoperative urodynamics. All women received preoperative prophylactic antibiotics (Kefzol® [Cefazolin] 2 g intravenously). They were operated on with a standardized technique by four experienced surgeons trained in this procedure. The technique is well described in our previous publication, with the difference that we did not use robotic assistance in this study [14]. We only used absorbable sutures (Vicryl™ Polyglactin 910 2-0 by Ethicon) or non-permanent tacks (AbsorbaTack™fixation device by Medtronic, Minneapolis, MN, USA) to fix the mesh to the vesico-vaginal fascia and always used the same polpyropylene titanized mesh (TiLOOP® “Prof Dubuisson” ® 9 × 41.5 cm, 65 g/m², pfm medical, Germany). Lateral arms were fixed to the abdominal peritoneum with absorbable tacks (AbsorbaTack™fixation device by Medtronic, Minneapolis, MN, USA). If a hysterectomy was required for associated myomas or adenomyosis, we performed a supracervical hysterectomy. In case of associated posterior compartment POP, we performed a standard posterior colporraphy. When urodynamically proven concomitant stress urinary incontinence (SUI) was present, women were offered to be treated during the same procedure by either Burch colposuspension or a midurethral sling. All women had a standardized follow-up examination by their surgeon at 4 to 6 weeks postoperatively. From July 2013 up to December 2018, all women were assessed by two of the authors (NV and KC) who were not the surgeons. A gynecological examination with POP-Q assessment was performed, and global satisfaction was assessed with the PGI-I questionnaire [15]. We collected variables including age, weight, height, parity, number of vaginal deliveries, menopausal status, the presence of diabetes, asthma, smoking, COPD, heart disease, constipation, sexual activity and history of a previous surgery for POP or urinary incontinence (UI). Preoperative, perioperative and long-term data were collected in a case report form for each woman.

Many authors agree that clinically relevant POPs are those that extend beyond the hymen [16, 17]. For this reason, we defined failure as POP beyond the hymen. We considered a subjective success rate of our medical care if the PGI was ≤ 2. Complications such as mesh exposure or mesh extrusion, de novo SUI, reoperation for SUI or recurrent POP, and de novo dyspareunia were systematically assessed.

We performed descriptive statistics by using a mean with standard deviation (SD) and the range to describe the general characteristics of our cohort. When more appropriate, we used a median with interquartile (IQ) range. Data analysis was managed using SPSS 25 statistical software (SPSS Inc., Chicago, IL, USA).

## Results

POP repair was performed successfully in all 88 patients without any intraoperative complication, in particular no hemorrhage or bladder or rectal injury. Nine women were lost to follow-up. The characteristics of the 79 women available for analysis are shown in Table 1. Mean age was 59.6 (SD 11.3) years ranging from 39.4 to 82.3 years. Mean BMI was 25.8 kg/m^2^ and mean follow-up 3.4 (SD 1.6) years. Ten women (12.7%) had a history of previous POP repair, and only one had previous stress urinary incontinence (SUI) surgery. Eight women (10.1%) had POP stage 2, 66 (83.5%) had stage 3, and 5 (6.3%) had stage 4. POP exceeded the hymen in 73 women (92.4%). Fifty-two women complained of posterior compartment prolapse symptoms and required concomitant posterior colporraphy. Thirty-eight patients (48.1%) had associated preoperative SUI, and occult incontinence was found in five additional patients during preoperative urodynamics (Table 2). Twenty-one patients (26.6%) decided to be treated, 7 (8.9%) with suburethral slings and 14 (17.7%) with Burch colposuspension. Among the 17 patients who decided not to be treated for SUI, 10 (58.8%) continued to complain of postoperative SUI, but only 1 decided to be treated with a suburethral sling 1 month after the POP operation. Eleven women (13.9%) required associated supracervical hysterectomy for benign diseases (myomas and adenomyosis). One of them had a history of previous POP repair involving cervical amputation. She developed infection of the mesh through a cervical fistula during the early postoperative period and required removal of the mesh 6 weeks later. There were otherwise no early postoperative complications, in particular no fever and no hematoma. The median duration of the full procedure, including associated interventions, was 180.0 (interquartile range [IQ] 180–240) min. The mean estimated blood loss was 87.5 (SD 114.8) ml (Table 3). The median postoperative stay was 3 (IQ range 2.0–4.0) days. Four patients (5.1%) were operated on for recurrence before assessment control. One woman had posterior colporraphy for stage 2 rectocele 20 months after the initial operation. One patient had sacrocolpopexy for stage 2 POP with the cervix beyond the hymen 13 months after LLS. Another had a subtotal hysterectomy with sacrocolpopexy for stage 3 uterine prolapse 19 months after the first operation, and the last one had stage 3 elytrocele and rectocele 34 months later also treated by sacocolpopexy. The objective cure rate (no prolapse beyond the hymen in any compartment and no reoperation for POP recurrence) was 87.3%. Tables 4 and 5 show the details on the anatomical results at short- and long-term follow-up. The subjective success rate of our medical care (PGI ≤ 2) was 96.2% at long-term follow-up (Table 6). There was no mesh exposure or extrusion. Two women (2.5%) reported de novo SUI and required further midurethral sling. One woman (1.3%) reported de novo dyspareunia.

**Table 1:** Characteristics of the study population (*N* = 79)

Characteristics	Value
Age (years) mean ± SD (range)	59.6 ± 11.1 (39.4–82.3)
Height (cm) mean ± SD (range)	161.1 ± 7.4 (143.0–176.0)
Weight (kg)	67.0 ± 11.7 (49.0–100.0)
BMI (kg/m^2^) mean (SD)	25.8 ± 4.0 (19.3–38.1)
Obese (BMI > 30), *n* (%)	13 (16.5%)
Caucasian	74 (93.7%)
Asian	3 (3.8)
South American	1 (1.3)
Parity mean ± SD (range)	2.2 ± 0.8 (1-5)
Nulliparous	3 (3.8%)
Primiparous	14 (17.7%)
Multiparous (≥ 2 deliveries)	62 (78.5%)
Number of vaginal deliveries mean (SD)	2.0 ± 0.9 (0-5)
Cesarean	5 (6.3%)
Menopause	56 (70.9%)
COPD, *n* (%)	0
Smoking > 5 cigarettes/day, *n* (%)	9 (11.4%)
Diabetes, *n* (%)	8 (10.1%)
Hypertension and cardiovascular disease, *n* (%)	4 (5.1%)
Constipation, *n* (%)	33 (41.8%)
Sexual activity, *n* (%)	52 (65.8%)
Previous hysterectomy, *n* (%)	7 (8.9%)
Previous POP surgery*	10 (12.7%)
Previous UI surgery, *n* (%)**	1 (1.3%)

Data are presented as mean (SD) and n (%)

SD = standard deviation; BMI = body mass index; COPD = chronic obstructive pulmonary disease

*Six anterior colporraphy and eight posterior colporraphy in eight women

**One TVT

**Table 2:** Preoperative pelvic floor characteristics of the study population

Characteristic	*N* = 79 (%)
POP-Q stage, *n* (%)	
Stage 1	0
Stage 2	8 (10.1)
Stage 3	66 (83.5)
Stage 4	5 (6.3)
POP beyond hymen	73 (92.4)
UI	
SUI	38 (48.1)
Pure SUI	28 (35.4)
Pure UUI	3 (3.8)
MUI	10 (12.7)
SUI, *n* (%)	
Grade 1	29 (36.7)
Grade 2	7 (8.9)
Grade 3	2 (2.5)
Occult stress incontinence at urodynamics, *n* (%)	5 (6.3)
Anal incontinence	2 (2.5)

SUI: stress urinary incontinence. UUI: urge urinary incontinence. MUI: mixed urinary incontinence

**Table 3:** Perioperative characteristics (*N* = 79)

Characteristic	Value
Operative time (min) median (25th to 75th percentile)	180.0 (180–240)
Concomitant procedures, *n* (%)	44 (55.7)*
Adhesiolysis, *n* (%)	15 (19.0)
Posterior colporraphy, *n* (%)	52 (65.8)
Supracervical hysterectomy, *n* (%)	11 (13.9)
SUI surgery, *n* (%)	21 (26.6)
Burch colposuspension	14 (17.7)
Suburethral sling	7 (8.9)
Bilateral prophylactic salpingectomy, *n*(%)	
Adnexectomy	
Bilateral	5 (6.3)
Unilateral, *n* (%)	4 (5.1)
Estimated blood loss (ml) mean ± SD (range)	87.5 ± 114.8 (0–500)
Intraoperative complication, *n* (%)	1 (1.3)**
Blood transfusion	0
Conversion to laparotomy, *n* (%)	0
Bladder injury	0
Bowel injury during laparoscopy, *n* (%)	0

IQ: interquartile

*One patient may have more than one associated procedure

**This was a superficial serous lesion of the rectum during posterior colporraphy

**Table 4:** Comparison between pre- and postoperative POP (*N* = 79)

Stage	Preoperative	Early postoperative (4 to 6 weeks)	Late postoperative* (at mean 3.4 years)
Stage 0	0	42 (53.2)	16 (20.3)
Stage 1	0	27 (34.2)	15 (19.0)
Stage 2	8 (10.1)	10 (12.7)	45 (57.0)
Beyond hymen	2 (2.5)	0	3 (3.8)
Stage 3	66 (83.5)	0	3 (3.8)
Stage 4	5 (6.3)	0	0
POP beyond hymen	73 (92.4)	0	6 (7.6)
Anatomical success rate (POP not exceeding hymen)		79 (100)	73 (92.4)

*Four patients reoperated for recurrence in between. Overall anatomical success: 87.3%

**Table 5:** Detailed anatomical results (*N* = 79)

Stage	Preoperative	Early postoperative (4 to 6 weeks)	Late postoperative* (at mean 3.4 years)
Anterior compartment (cystocele)			
0	0	49 (62)	24 (30.4)
1	3 (3.8)	24 (30.4)	27 (34.2)
2	16 (20.3)	6 (7.6)	28 (35.4)
Beyond hymen	1	0	1
3	57 (72.2)	0	0
4	3 (3.8)	0	0
Middle compartment (uterine or vaginal vault prolapse)			
0	5 (6.3)	64 (81)	57 (72.2)
1	11 (13.9)	13 (16.5)	14 (17.7)
2	32 (40.5)	2 (2.5)	6 (7.6)
Beyond hymen	1	0	2
3	27 (34.2)	0	2 (2.5)
4	4 (5.1)	0	0
Posterior compartment (rectocele and or elytrocele)			
0	13 (16.5)	59 (74.7)	36 (45.6)
1	25 (31.6)	16 (20.3)	18 (22.8)
2	38 (48.1)	4 (5.1)	24 (30.4)
Beyond hymen	0	0	0
3	3 (3.8)	0	1 (1.3)
4	0	0	0

*Four patients reoperated for recurrence in between

**Table 6:** Subjective outcome (PGI-I)

PGI-I score	Long term (mean 3.4 years) *N* = 79
PGI-I ≥ 2	76 (96.2)
1: Very much better	61 (77.2)
2: Much better	15 (19.0)
3: A little better	1 (1.3)
4: No change	1 (1.3)
5: A little worse	1 (1.3)
6: Much worse	0
7: Very much worse	0

Results are presented as *n* (%)

## Discussion

Our study confirms the safety and effectiveness of LLS to treat anterior and apical POP with excellent anatomical and functional outcomes after a mean follow-up of > 3 years. Fixation of the mesh to the vesicovaginal fascia with absorbable material does not affect long-term support. When used in association with macroporous polypropylene mesh, the exposure/extrusion rate is zero in our study. Given the current controversy surrounding the usage of mesh, this is a very reassuring result. In the first series of LLS by Dubuisson et al., the exposure/extrusion rate was close to 6% [9]. In comparison, mesh exposure/extrusion rates in contemporary sacrocolpopexy studies vary between 3 and 10% [18, 19]. Dubuisson used different types of meshes made of polyester and later on of polypropylene and fixed them to the vesico-vaginal fascia with permanent polyester sutures or glue. Some patients also had mesh placed in the rectovaginal space. We showed in a recent report that type 3 polyester mesh (macroporous with either multifilamentous or microporous components) and a posterior mesh placement increased the risk of mesh exposure [12]. In the present study, we only used type 1 macroporous polypropylene mesh and treated the posterior compartment vaginally with native tissue if necessary*.* This strategy is sustained by a recent Cochrane review showing that the posterior compartment is best treated by vaginal access without mesh [11]. In a recent LLS series of 120 women with a 2-year follow-up, Mereu et al. used the same mesh as in our study, but fixed it to the vesico-vaginal fascia with permanent polyester sutures [20]. The exposure/extrusion rate was 0.8%. We believe the use of polyester permanent sutures in itself may increase the risk of mesh-related complications by conducting bacteria from the vagina to the mesh, especially if sutures are too close to the vaginal mucosa. This was demonstrated in a recent large series of sacrocolpopexy in which patients presented with extrusion of non-dissolvable polyester sutures and later required the removal of an exposed prosthesis in the vagina [21]. In Simmoncini et al.'s robotically assisted LLS (RALLS) study, they used long-term absorbable synthetic sutures to secure the mesh to the vaginal wall [10]. They observed immediate extrusion of one suture and had to remove it with 1 cm^2^ of the underlying mesh a few days after the initial surgery. Therefore, the use of short-term absorbable sutures to fix the mesh to the vesicovaginal fascia may be advisable. It may reduce the risk of mesh-related complications without impairing results, as confirmed by the present study. Fibrosis forms early on and is enough from our point of view to secure the mesh to the vaginal wall.

LLS lends itself perfectly to uterine preservation, which today is an important element for many women [22]. Moreover, avoiding hysterectomy makes the procedure faster and limits the risk of potential complications. In addition, contrary to what we thought in the past, uterus preservation during POP repair does not seem to alter long-term results [23]. In LLS, the lateral arms of the mesh are suspended retroperitoneally under the round ligaments following the lateral attachment of the uterus, which results in a very natural anatomic suspension of the apical compartment. In comparison, during sacrocolpopexy, there is a slight posterior and lateral traction to the right. Preserving the uterus during POP surgery not only reduces operating time and perioperative risks, but also reduces long-term risk of mesh erosion in case of total hysterectomy [24, 25]. In the present study, 11 women underwent supracervical hysterectomy for symptomatic myomas or adenomyosis but most of our patients (77.2%) had hysteropexy. The only early postoperative complication of this cohort occurred in a woman with a history of previous POP surgery with cervical amputation. It can be hypothesized that the cervical canal was probably too short and no longer competent to provide an effective barrier against vaginal bacteria proliferation into the overlying mesh. During the early postoperative period, she developed a cervical fistula and infection of the mesh requiring its removal. As with total hysterectomy, we believe mesh should not be used after previous cervical amputation in case of required supracervical hysterectomy, and an alternative POP repair without mesh should be preferred. Based on these observations, we believe the uterus should be preserved as much as possible in most cases of POP surgery.

The cure rate was 87.3% in our study with a reoperation rate for recurrence in 5.1% for a mean follow-up of 3.4 years. In Dubuisson's series, with a shorter follow-up of 18 months, the success rate was 86% with a reoperation rate for recurrence of 4.6% [9]. In Mereu et al.'s LLS series, 89% of women were asymptomatic at 2-year follow-up, and repeat surgery for POP occurred in 6.4% of cases, which is very similar to our study [20]. Contrary to these two studies, we only used absorbable material to secure the mesh to the vesico-vaginal fascia. This did not alter the results, which supports the hypothesis developed above that it is not useful to fix the prostheses with non-absorbable material, as the fibrosis created by the scarring process alone is sufficient to provide support. The results of this study are also very similar to those of laparoscopic sacrocolpopexy (LSCP). In Sarlos et al.'s LSCP series with 5-year follow-up, the success rate was 83.8% with a reoperation rate for recurrence of 3.5%. Like Dubuisson and Mereu, Sarlos et al. used non-absorbable polyester sutures (Ethibon ®) to fix a macroporous polypropylene mesh (Gynemesh®) and had a 2.9% mesh exposure rate [19]. In the series of Rivoire et al. using a very similar technique, the success rate at 33 months mean follow-up was also 89% with a 5% extrusion/exposure rate [4].

Concerning intraoperative complications, LLS seems safer than LSCP. There were no intraoperative complications in our study or in the Mereu et al. study [20]. In comparison, Sarlos et al. using LSCP reported three cases of rectal injury with two requiring laparotomy and one with septical peritonitis. They also reported four bladder lesions and one mechanical ileus requiring laparotomy [3]. In Rivoire et al.'s LSCP study, they reported one hemorrhage, but no conversion to laparotomy; however, one patient had spondylodiscitis [4]. Peri- and intraoperative complications such as presacral hemorrhage are serious complications of LSCP, which can be avoided with LLS. In Cosson et al.'s series of 77 LSCPs, the main operative complications included one rectal injury and three bleeding complications requiring reoperations, with 1 perioperative hemorrhage requiring conversion to laparotomy [26]. Weinberg et al., in a large retrospective cohort study with 7232 LSCPs, identified 5.5% serious complications including sepsis, cardiac arrest and death [27]. Ross and Nehzat in their avant-garde work already reported similar injuries after LSCP [28, 29]. For all these reasons, we believe LLS to be safer than LSCP.

In Sarlos et al.'s LSCP study they also described a 24% de novo SUI rate at 12 months increasing to 32% at 60 months follow-up [19]. This was not the case after LLS where Mereu et al. and we had only 2.5% de novo SUI [20]. Lateral suspension follows round ligaments' direction and therefore the natural anatomical support of the uterus and vaginal apex. By improving anterior vaginal fascia support, it might even help to correct SUI. In our study, we observed that among the 17 patients suffering from preoperative SUI who decided not to be treated concomitantly, 7 of them (41.2%) were spontaneously cured postoperatively. This was also found in the Martinello and al.'s recent LLS series where 44% of women complained of SUI to some degree preoperatively but only 19% postoperatively, without any SUI surgery [30]. This might be another advantage over LSCP, but these results need to be confirmed by further studies.

A limitation of this study is the small number of patients. Larger series are required to confirm these very encouraging results. A few patients were not available for follow-up probably because of change of address or immigration. They might have been followed and reoperated outside our university clinic resulting in an underestimation of recurrence or complication rate. However, this is very unlikely as our clinic is the only public hospital in the canton of Geneva. Women followed in public hospitals in Switzerland rarely go to private clinics because of their lack of private health insurance coverage, and Swiss health insurances only exceptionally permit a patient to be operated in another canton or country.

The strengths of this study are its prospective character with systematic pre- and postoperative assessment using the POP-Q system and a standardized surgical technique. In addition, the postoperative status assessment was carried out by two investigators who were not the surgeons, thus limiting the risk of assessment bias.

## Conclusion

LLS with a macroporous polypropylene mesh secured to the vesico-vaginal fascia with non-permanent sutures is a safe and effective procedure to treat anterior and apical POP. The risk of mesh-related complications is very low, and the procedure may be associated with improvement of pre-existing SUI. We believe that this technique represents a good alternative to LSCP, especially in case of uterus preservation. It has potential advantages, with reduction of potential serious complications, and deserves to be included in the therapeutic arsenal of the modern urogynecologist.

## Abbreviations

LLS: Laparoscopic lateral suspension
RALLS: Robotically assisted laparoscopic lateral suspension
LSCP: Laparoscopic sacrocolpopexy
POP: Pelvic organ prolapse
POP-Q: Pelvic organ prolapse quantification
SUI: Stress urinary incontinence
UI: Urinary incontinence
PGI-I: Patient global impression of improvement
BMI: Body mass index
SD: Standard deviation
UI: Urinary incontinence
IQ: Interquartile
